# Neuronal C‐Reactive Protein/Fc*γ*RI Positive Feedback Proinflammatory Signaling Contributes to Nerve Injury Induced Neuropathic Pain

**DOI:** 10.1002/advs.202205397

**Published:** 2023-02-02

**Authors:** Fan Liu, Li Zhang, Si Su, Yehong Fang, Xiang‐sha Yin, Huan Cui, Jianru Sun, Yikuan Xie, Chao Ma

**Affiliations:** ^1^ National Human Brain Bank for Development and Function Department of Human Anatomy Histology and Embryology Neuroscience Center Institute of Basic Medical Sciences Chinese Academy of Medical Sciences School of Basic Medicine Peking Union Medical College Beijing 100005 P. R. China; ^2^ Department of Anesthesiology Beijing Friendship Hospital Capital Medical University Beijing 100050 P. R. China; ^3^ Chinese Institute for Brain Research Beijing 102206 P. R. China

**Keywords:** CD64, C‐reactive protein, dorsal root ganglion, Fc‐gamma receptor I, spleen tyrosine kinase

## Abstract

Neuropathic pain is difficult to treat in clinical practice, and the underlying mechanisms are insufficiently elucidated. Previous studies have demonstrated that the neuronal Fc‐gamma‐receptor type I (Fc*γ*RI) of the dorsal root ganglion (DRG) mediates antigen‐specific pain. However, the mechanisms of neuronal Fc*γ*RI in neuropathic pain remain to be explored. Here, it is found that the activation of Fc*γ*RI‐related signals in primary neurons induces neuropathic pain in a rat model. This work first reveals that sciatic nerve injury persistently activates neuronal Fc*γ*RI‐related signaling in the DRG, and conditional knockout (CKO) of the Fc*γ*RI‐encoding gene *Fcgr1* in rat DRG neurons significantly alleviates neuropathic pain after nerve injury. C‐reactive protein (CRP) is increased in the DRG after nerve injury, and CRP protein of the DRG evokes pain by activating neuronal Fc*γ*RI‐related signals. Furthermore, microinjection of naive IgG into the DRG alleviates neuropathic pain by suppressing the activation of neuronal Fc*γ*RI. These results indicate that the activation of neuronal CRP/Fc*γ*RI‐related signaling plays an important role in the development of neuropathic pain in chronic constriction injury (CCI) rats. The findings may provide novel insights into the neuroimmune responses after peripheral nerve injury and suggest potential therapeutic targets for neuropathic pain.

## Graphical abstract

The study shows that Fc‐gamma‐receptor type I (Fc*γ*RI) is expressed in neurons of the dorsal root ganglion and contributes to neuroinflammation via Syk signaling. In addition, C‐reactive protein (CRP) activates neuronal Fc*γ*RI/Syk‐related signals. Targeting CRP/Fc*γ*RI/Syk may be effective for the treatment of neuropathic pain.

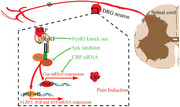

## 1 Introduction

Neuropathic pain is the most common form of chronic pain resulting from damage or disease of the somatosensory system, symptoms of which include spontaneous pain, hyperalgesia, and allodynia.^[^ ^1^ ^]^ Neuropathic pain, one of the main problems troubling human health, not only seriously affects patients’ quality of life but also places a huge financial burden on society. A systematic review of epidemiological studies reported that the prevalence of neuropathic pain has been estimated at 6.9–10.0% within the general population, and the number of neuropathic pain patients is increasing year by year.^[^ ^2^ ^]^ Because the pathogenesis of neuropathic pain is still unclear, the analgesic effects of existing treatments and drugs are not satisfactory and are often coupled with intolerable side effects. Therefore, it is urgent to study and elucidate the occurrence mechanism and pathogenesis of neuropathic pain and to find new therapeutic strategies and drug targets on this basis.

In previous research reports, the immune system and peripheral nervous system play an important role in neuropathic pain. Research has found that neuropathic pain is characterized by immune diseases.^[^ ^3^ ^]^ Peripheral nerve injury often results in the disintegration of nerve fibers, induces inflammation, and may release autoantigens.^[^ ^4^ ^]^ In recent years, it has been reported that peripheral nociceptive neurons have immune sensing functions and immune recognition through the expression of immune‐related receptors such as Fc‐gamma receptors (Fc*γ*Rs), Fc‐epsilon receptors (Fc*ε*Rs), and Toll‐like receptors.^[^ ^5^ ^]^ Our previous research revealed that partial neurons of the DRG and trigeminal ganglion in rodents express Fc*γ*RI (a type of receptor with high affinity activation for IgG) and can be activated by the IgG‐immune complex (IgG‐IC) to lead to pain and hyperalgesia.^[^ ^5^, ^6^ ^]^ DRG neurons not only expressed Fc*γ*RI (coexpressed with markers of nociceptors such as transient receptor potential cation channel subfamily V member 1 (TRPV1), isolectin B4 (IB4), calcitonin gene‐related peptide (CGRP), and substance P) but could also be activated by IgG‐IC in vitro or in vivo.^[^ ^5^, ^6^ ^]^ However, whether neuronal Fc*γ*RI in the DRG, which is activated by ligands, contributes to neuropathic pain has not been fully elucidated.

In the present study, we explored the potential roles of neuronal Fc*γ*RI in the DRG after nerve injury. We hypothesized that Fc*γ*RI‐related signals in DRG neurons play a role in the development of neuropathic pain after nerve injury. In this study, we tested this hypothesis using an *Fcgr1* conditional knockout (CKO) rat model of chronic constriction injury (CCI) of the sciatic nerves.

## 2 Results

### 2.1 Neuronal Fc*γ*RI is Upregulated in the DRG After Peripheral Nerve Injury

**Figure 1:**
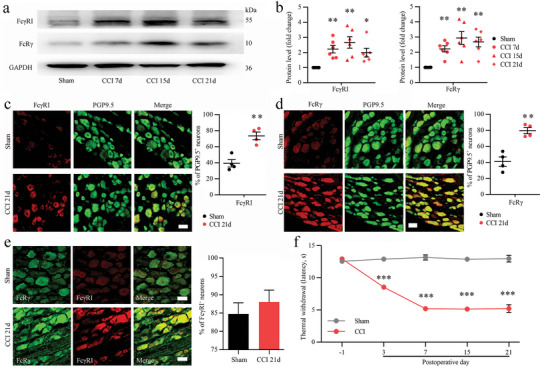
Expression and cellular distributions of Fc‐gamma‐receptor type I (Fc*γ*RI) and Fc receptor gamma‐chain (FcR*γ*) in rat dorsal root ganglions (DRGs) after chronic constriction injury (CCI) surgery. a,b) Western blot analysis (a) and quantification of the protein levels of Fc*γ*RI and FcR*γ* (b) in DRGs from sham, CCI 7 d, CCI 15 d and 21 d rats after surgery. *n* = 6 per group, ^*^ *p* < 0.05, ^**^ *p* < 0.01 versus the sham group, one‐way ANOVA. c) Double immunofluorescence showing the cellular distribution of Fc*γ*RI (red) and the neuronal marker PGP9.5 (green) in DRGs from sham and CCI 21 d rats after surgery. d) Double immunofluorescence showing the cellular distribution of FcR*γ* (red) with PGP9.5 (green) in DRGs from sham and 21 d rats after surgery. e) Double immunostaining showing cellular colocalization of Fc*γ*RI (red) and FcR*γ* (green) in rat DRGs from the sham and CCI 21 d groups. In c–e, scale bars = 50 µm, *n* = 4 per group, ^**^ *p* < 0.01 versus the sham group by Student's *t* test. f) CCI‐induced thermal hypersensitivity manifested as a lowered threshold of thermal withdrawal. *n* = 10–26 per group, ^***^ *p* < 0.001 versus the sham group by two‐way ANOVA.

Previous studies demonstrated that neuronal Fc*γ*RI in the DRG could be directed by IgG‐IC and mediate joint pain in animal models of rheumatoid arthritis (RA).^[^ ^5^, ^6^, ^7^ ^]^ Peripheral nerve injury induced protein changes in DRGs play a critical role in neuropathic pain generation. CCI of the rat sciatic nerve is a well‐characterized animal model of neuropathic pain.^[^ ^8^ ^]^ First, we examined the protein expression of Fc*γ*RI and Fc receptor gamma‐chain (FcR*γ*) in the nerve‐injured DRGs of CCI rats. Our Western blot analysis showed that CCI induced a long‐lasting increase in Fc*γ*RI and FcR*γ* protein levels in the nerve‐injured DRGs of rats (**Figure** **1**a,b). The mRNA levels of *Fcgr1* and *Fcrg* in the DRG were also detected by qPCR. *Fcgr1* and *Fcrg* mRNA levels were continuously increased after nerve injury in the DRG of rats (Figure S1a,b, Supporting Information). To determine the cellular distribution of Fc*γ*RI and FcR*γ*, we then performed double immunolabeling using PGP9.5 (neuronal marker) with Fc*γ*RI or FcR*γ*. Immunostaining showed that Fc*γ*RI and FcR*γ* were predominantly immunoreactive in DRG neurons (PGP9.5‐positive cells); after CCI surgery, Fc*γ*RI and FcR*γ* expression in DRG neurons was significantly upregulated compared with that in DRG neurons in the sham group (Figure 1c,d). Meanwhile, double immunostaining revealed that the FcR*γ* protein was mainly coexpressed in Fc*γ*RI‐positive cells, and the coexpression ratio of FcR*γ* with Fc*γ*RI in DRG neurons was not significantly changed in the sham or CCI surgery group (Figure 1e). Figure 1f shows that the CCI operation induced rapid and persistent thermal hyperalgesia, which detected a significant decrease in the paw from 3 to 21 days after the CCI operation. Compared with the sham group, rats with CCI also showed a decrease in paw mechanical withdrawal threshold at 3 days after the CCI operation, which was maintained throughout the 21 days after the CCI operation (Figure S1c, Supporting Information).

### 2.2 Neuronal Syk Signaling is Activated in the DRG after Peripheral Nerve Injury

**Figure 2:**
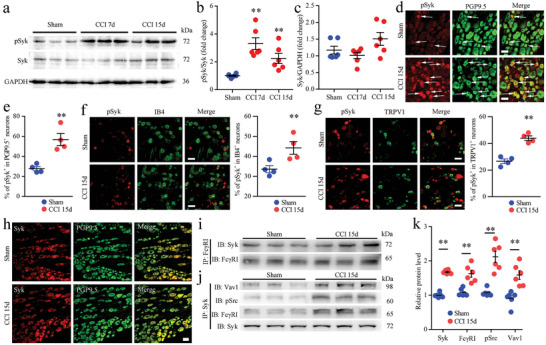
Expression and cellular distributions of Syk in rat dorsal root ganglions (DRGs) after chronic constriction injury (CCI) surgery. a–c) Western blot analysis (a) and quantification of the protein levels of phosphorylated Syk (pSyk) (b) and Syk (c) in DRGs from sham, CCI 7 d and 15 d rats after surgery. *n* = 6 per group, ^**^ *p* < 0.01 versus the sham group, one‐way ANOVA. d) Double immunofluorescence showing the cellular distribution of pSyk (red) with the neuronal marker PGP9.5 (green) in DRGs from sham and CCI rats 15 d after surgery. e) Proportion of pSyk‐positive neurons in the DRGs of sham and CCI rats 15 d after surgery. f,g) Double immunostaining showing coexpression of pSyk with IB4 (f) and TRPV1 (g) in DRGs from sham and CCI rats 15 d after surgery. h) Immunofluorescence staining showing the cellular distribution of Syk (red) with the neuronal marker PGP9.5 (green) in DRGs from sham and CCI rats 15 d after surgery. i) Coimmunoprecipitation (Co‐IP) showed the interaction between Fc‐gamma‐receptor type I (Fc*γ*RI) and Syk in DRG tissue from sham and CCI rats 15 d after surgery. j) Co‐IP showed the interaction between Syk and Vav1, phosphorylated Src (pSrc), and Fc*γ*RI in DRG tissue from sham and CCI rats 15 d after surgery. k) Quantification of Co‐IP of Syk, Fc*γ*RI, pSrc, and Vav1 in DRGs from sham and CCI rats 15 d after surgery. *n* = 6 per group, ^**^ *p* < 0.05 versus the sham group, Student's *t* test. Scale bars: 30 µm (d), 50 µm (f–h). f,g) ^**^ *p* < 0.01 versus sham, Student's *t* test, *n* = 4 per group. Arrows show positive neurons.

To determine whether increased Fc*γ*RI and FcR*γ* expression levels are associated with increased related signaling activity, we measured the level of spleen associated tyrosine kinase (Syk), which is coupled with Fc*γ*RI.^[^ ^9^ ^]^ Syk is a member of the family of nonreceptor‐type Tyr protein kinases.^[^ ^9b^ ^]^ It, which is widely expressed in immune cells, is involved in coupling activated immunoreceptors such as Fc*γ*Rs and Fc*ε*RI to downstream signaling events that mediate receptor activation.^[^ ^9b^ ^]^ Syk is required for Fc*γ*RI‐induced excitation of sensory neurons in the DRG.^[^ ^10^ ^]^ Therefore, we examined the expression, activation, and cellular distribution of Syk in the DRG after CCI surgery. The experimental results indicated that phosphorylated Syk (pSyk) was expressed at higher levels than sham surgery in the DRGs of CCI rats (**Figure** **2**a,b). The total Syk protein level of the rat DRGs was not changed after CCI surgery (Figure 2a,c). Double immunofluorescence images showed that pSyk was mainly expressed in the small neurons of the DRG and was significantly higher in DRG neurons after CCI surgery (Figure 2d,e). Furthermore, double immunofluorescence revealed that pSyk was coexpressed with the nociceptive neuronal markers IB4 and TRPV1 in DRG neurons (Figure 2f,g). In addition, we further revealed that the total Syk of neurons was not significantly changed in rat DRGs after CCI surgery (Figure 2h). Immunostaining also indicated that Syk protein was coexpressed with Fc*γ*RI in the DRG neurons of rats (Figure S1d, Supporting Information). The fluorescence in situ hybridization revealed that *Syk* mRNA colocalized with *Fcgr1* mRNA in DRG neurons (Figure S1e, Supporting Information).

Furthermore, we detected the relationship of Syk‐related proteins with Fc*γ*RI in DRG tissue after nerve injury. Coimmunoprecipitation (Co‐IP) revealed the presence of Syk with Fc*γ*RI in the same complex of DRG tissue (Figure 2i). We found increased recruitment of Syk to Fc*γ*RI after nerve injury (Figure 2k). Further Co‐IP showed that the proteins Fc*γ*RI, phosphorylated Src (pSrc), and Vav1 were related to the Syk protein and were more strongly bound to Syk proteins (Figure 2j,k).

**Figure 3:**
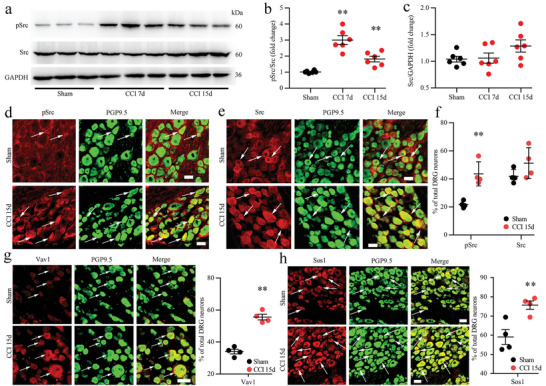
Expression, activation, and cellular distributions of Fc‐gamma‐receptor type I (Fc*γ*RI) related downstream signaling proteins in rat dorsal root ganglions (DRGs) after chronic constriction injury (CCI) surgery. a–c) Western blot analysis (a) and quantification of the protein levels of phosphorylated Src (pSrc) (b) and Src (c) in DRGs from sham, CCI 7 d and 15 d rats after surgery. *n* = 6 per group, ^**^ *p* < 0.01 versus the sham group, one‐way ANOVA. d,e) Double immunofluorescence showing the cellular distribution of pSrc (red, d) and Src (red, e) with the neuronal marker PGP9.5 (green) in DRGs from sham and CCI rats 15 d after surgery. f) Proportion of pSrc‐ and Src‐positive neurons in DRGs from sham and CCI rats 15 d after surgery. g) Double immunofluorescence showing the cellular distribution and positive rate of Vav1 in DRGs from sham and CCI rats 15 d after surgery. h) Immunofluorescence staining showing the cellular distribution and positive rate of Sos1 in DRGs from sham and CCI rats 15 d after surgery. Scale bars: 50 µm (d,e,g,h). ^**^ *p* < 0.01 versus sham, Student's *t* test, *n* = 4 per group (f,g,h). Arrows show positive neurons.

Western blot results also showed that CCI caused increased expression of pSrc in nerve‐injured DRGs and that total Src did not significantly change in DRGs after CCI (**Figure** **3**a–c). Similar to Syk, Src was also mainly distributed in small neurons, and pSrc‐immunopositive DRG neurons were more significantly increased in nerve‐injured DRGs (Figure 3d–f). Next, we observed that the Vav1 and Sos1 proteins, which are proinflammatory proteins downstream of Syk signaling, were also expressed and colocalized with PGP9.5 in the DRG and increased expression after CCI in the nerve‐injured DRGs using double immunofluorescence (Figure 3g,h).

### 2.3 Inhibition of Neuronal Fc*γ*RI‐Syk Signaling Mitigates Chronic Pain

**Figure 4:**
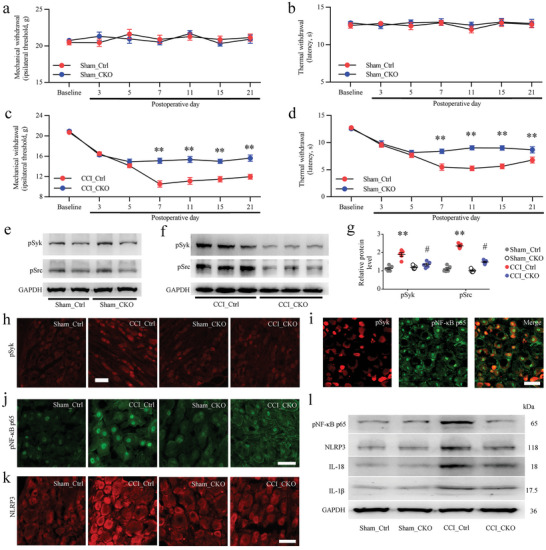
Neuronal Fc‐gamma‐receptor type I (Fc*γ*RI) mediated nerve injury induced pain and Syk‐related signaling activation. a,b) Comparison of the von Frey (a) and heat (b) withdrawal thresholds between *Fcgr1* conditional knockout (CKO) and littermate control (Ctrl) rats after sham operation, *n* = 9 per group, two‐way ANOVA. c,d) Nerve injury induced mechanical allodynia (c) and thermal hyperalgesia (d) were attenuated in CKO rats. *n* = 9 per group, two‐way ANOVA, ^**^ *p* < 0.01, CKO versus Ctrl. e,f) Western blot showing the expression levels of phosphorylated Syk (pSyk) and phosphorylated Src (pSrc) in Ctrl and CKO rat dorsal root ganglions (DRGs) after sham (e) and chronic constriction injury (CCI) (f) 21 d after injury. g) Data summary of the expression levels of pSyk and pSrc in Ctrl and CKO rat DRGs after sham and CCI 21 d after injury. One‐way ANOVA, *n* = 5 per group, ^**^ *p* < 0.01 versus the sham group of Ctrl rats; ^#^ *p* < 0.05 versus the CCI group of CKO rats. h) Immunofluorescence staining showing the pSyk cellular distribution in sham and CCI 21 d DRGs from Ctrl and CKO rats. i) Immunofluorescence staining showing cellular colocalization of pSyk (red) and phosphorylated NF‐kappa B p65 subunit (pNF‐*κ*B p65) (green) in the DRGs of rats 21 d after CCI surgery. j,k) Immunofluorescence staining showing pNF‐*κ*B p65 (j) transport into the nucleus and NLRP3 (k) expression in sham and CCI DRGs from Ctrl rats and CKO rats 21 d after surgery. Scale bars: 50 µm (h,i,j,k), *n* = 4 per group. l) Western blot showing the expression levels of pNF‐*κ*B p65, NLRP3, IL‐18, and IL‐1*β* in Ctrl and CKO rat DRGs after sham and CCI 21 d after injury.

CCI caused a substantial increase and activation of neuronal Fc*γ*RI/Syk signaling in the DRG. We continued to investigate the possible role in neuropathic pain. To determine the role of neuronal Fc*γ*RI in the development of nerve injury induced neuropathic pain in the CCI model, we selectively deleted *Fcgr1* in DRG neurons by crossing rats carrying *loxP*‐flanked *Fcgr1* with a primary sensory neuron–specific Cre line (*Pirt^Cre/+^)* to create *Fcgr1* CKO rats (Figure S2, Supporting Information). Compared with baseline, littermate control rats and CKO rats that underwent sham surgery did not show a decrease in the paw mechanical withdrawal threshold or thermal withdrawal latency (**Figure** **4**a,b). However, the results of pain‐related behaviors in CKO rats showed that both nerve injury induced mechanical allodynia and thermal hyperalgesia were alleviated compared to littermate control rats (Figure 4c,d).

To directly determine whether Syk signaling pathways are involved in neuronal Fc*γ*RI‐dependent neuropathic pain, we measured the effects of neuronal *Fcgr1* CKO of DRGs on the phosphorylation of Syk and Src in CCI rats. Western blot results showed that the increased pSyk and pSrc levels in nerve‐injured DRGs were significantly attenuated in the CKO rats after CCI (Figure 4e–g). Immunostaining showed that nerve injury induced pSyk‐immunopositive DRG neurons were significantly reduced in *Fcgr1* CKO rats (Figure 4h; Figure S3a, Supporting Information). Further research found that phosphorylated NF‐kappa B p65 subunit (pNF‐*κ*B p65) was predominantly coexpressed with pSyk in DRG neurons after CCI (Figure 4i). In addition, immunostaining and Western blot results revealed that DRG neuron *Fcgr1* conditional deletion significantly reduced the nuclear transport and phosphorylation of nuclear factor NF‐*κ*B p65 and the expression of NLR family pyrin domain containing 3 (NLRP3) in DRG neurons (Figure 4j–l; Figure S3b–e, Supporting Information). Furthermore, the nerve injury induced upregulation of IL‐1*β* and IL‐18 was significantly decreased in the DRGs of CKO rats (Figure 4l; Figure S3f,g, Supporting Information).

**Figure 5:**
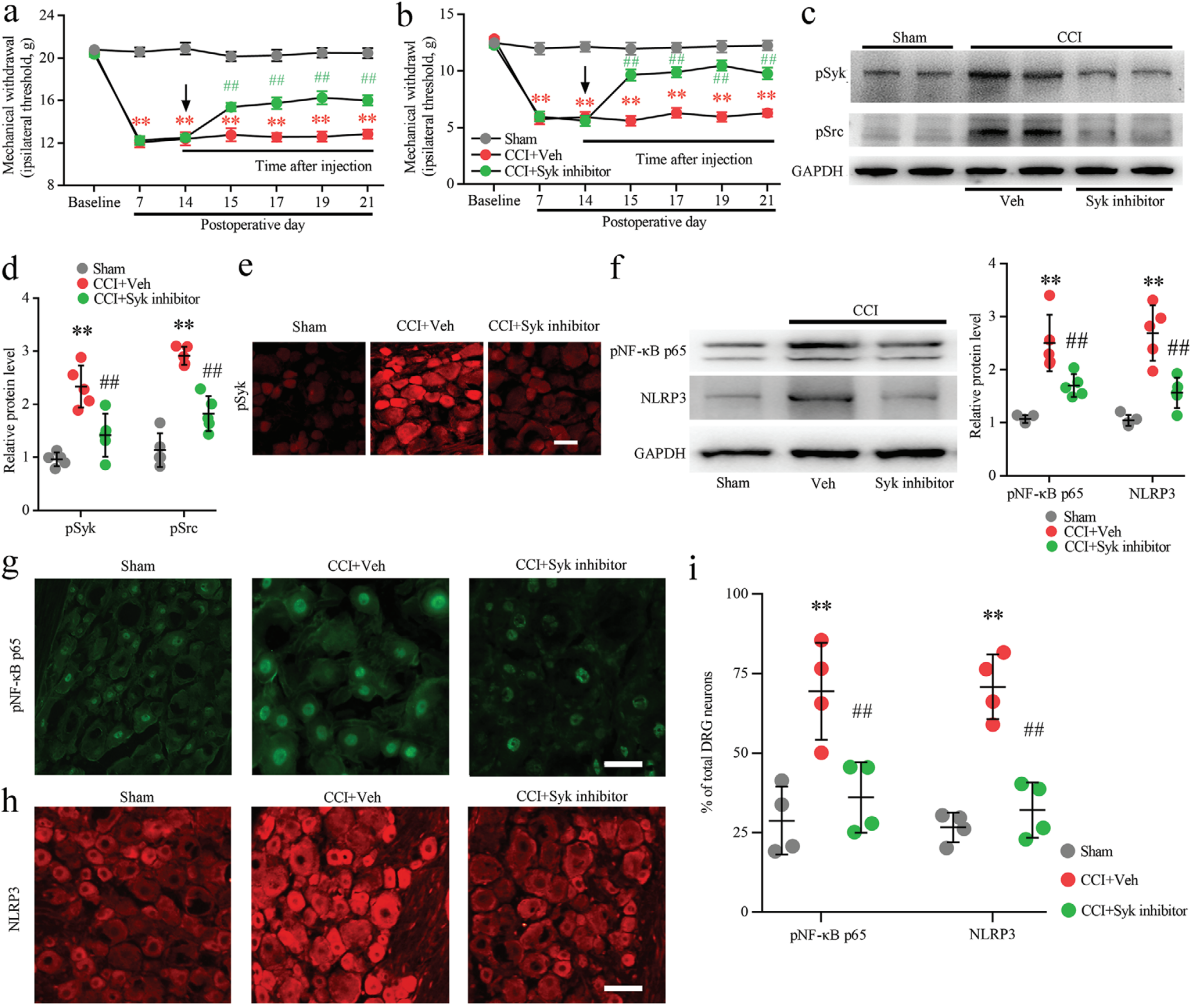
Inhibiting Syk activation attenuated nerve injury induced pain and NF‐*κ*B/NLR family pyrin domain containing 3 (NLRP3) signaling activation in the dorsal root ganglion (DRG). a,b) Chronic constriction injury (CCI) induced mechanical allodynia (a) and thermal hyperalgesia (b) were attenuated in rats by a Syk inhibitor (*n* = 9 per group). The first administration is indicated by an arrow on day 14 after CCI surgery. Rats were orally administered Syk inhibitor or vehicle b.i.d. from 14 to 21 d after CCI surgery. Two‐way ANOVA, ^**^ *p* < 0.01, CCI + Veh versus sham or CCI + Syk inhibitor; ^##^ *p* < 0.01, CCI + Syk inhibitor versus CCI + Veh or Sham. c) Western blot showing the activation levels of phosphorylated Syk (pSyk) and phosphorylated Src (pSrc) in DRGs from sham, CCI + Veh and CCI + Syk inhibitor rats 21 d after the operation. d) Data summary of the expression levels of pSyk and pSrc in the inhibitory effects of a Syk inhibitor on nerve injury induced rat DRGs after sham and CCI injury. ^**^ *p* < 0.01 versus the sham group; ^##^ *p* < 0.01 versus the CCI + Veh group, by one‐way ANOVA, *n* = 5 per group. e) Immunofluorescence staining showing the inhibitory effects of the Syk inhibitor on pSyk cellular distribution in DRGs 21 d after sham and CCI surgery. f) Western blot showing the inhibitory effects of the Syk inhibitor on nerve injury induced activation of phosphorylated NF‐kappa B p65 subunit (pNF‐*κ*B p65) and NLRP3 in DRGs. Data summary of the expression levels of pNF‐*κ*B p65 and NLRP3 in the inhibitory effects of a Syk inhibitor on nerve injury induced rat DRGs after sham and CCI injury. ^**^ *p* < 0.01 versus the sham group; ^##^ *p* < 0.01 versus the CCI + Veh group, by one‐way ANOVA, *n* = 5 per group. g,h) Immunofluorescence staining showing the inhibitory effects of the Syk inhibitor on pNF‐*κ*B p65 (g) and NLRP3 (h) cellular distribution in DRGs 21 d after sham and CCI surgery. i) The positive rates of pNF‐*κ*B p65 and NLRP3 in DRGs from sham, CCI + Veh and CCI + Syk inhibitor rats 21 d after the operation. ^**^ *p* < 0.01 versus the sham group; ^##^ *p* < 0.01 versus the CCI + Veh group, by one‐way ANOVA, *n* = 4 per group. Scale bars: 50 µm (e,g,h).

In the CCI model of CKO rats, the activation of neuronal Syk signaling pathways was alleviated in the DRG. To further investigate the potential function of Syk signaling in nerve injury induced neuropathic pain, we delivered a Syk inhibitor (PRT062607 (P505‐15) HCl) or vehicle (normal saline) to rats on postoperative day (POD) 14 (orally twice a day). We found that Syk inhibitor administration daily for 7 consecutive days (from POD14 to POD21) produced long‐lasting alleviation of nerve injury induced mechanical allodynia and thermal hyperalgesia in rats (**Figure** **5**a,b). To determine whether Syk/Src mediated neuronal Fc*γ*RI signaling, we detected the nerve injury induced activation of Syk/Src signaling in the DRGs of CCI rats after Syk inhibitor administration. Our results showed that nerve injury induced pSyk and pSrc expression in the DRG was significantly reduced after Syk inhibitor administration (Figure 5c–e). Meanwhile, the Western blot and immunostaining results revealed that nerve injury induced activation of NF‐*κ*B p65 and upregulation of NLRP3 in neurons were also alleviated in the DRGs after Syk inhibitor administration (Figure 5f–i).

### 2.4 Neuronal CRP Induced Pain and Neuroinflammation via Fc*γ*RI

**Figure 6. C:**
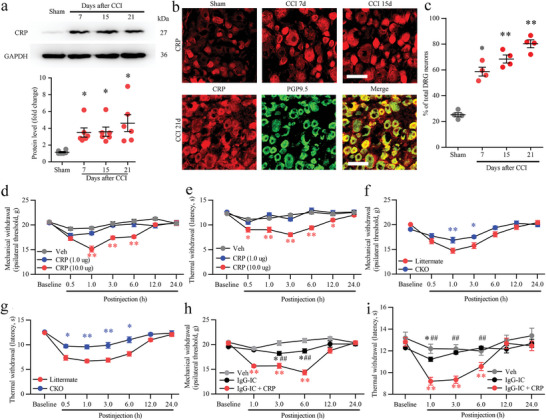
hronic constriction injury (CCI) increased C‐reactive protein (CRP) expression in the dorsal root ganglions (DRGs) and CRP‐induced pain in rats. a) Western blot analysis and quantification of CRP protein levels in DRGs from sham, CCI 7 d, 15 d, and 21 d rats after surgery. One‐way ANOVA, *n* = 6 per group, ^*^ *p* < 0.05 versus the sham group. b) Immunostaining of CRP in the DRGs of sham and CCI 7 d, CCI 15 d, and CCI 21 d rats. c) The positive rates of CRP in DRG neurons from sham, CCI 7 d, CCI 15 d, and CCI 21 d rats. Scale bars: 50 µm. One‐way ANOVA, *n* = 4 per group, ^*^ *p* < 0.05, ^**^ *p* < 0.01 versus the sham group. d,e) CRP induced mechanical (d) and thermal (e) hyperalgesia in naive rats. Changes in the paw withdrawal mechanical threshold (e) and the paw withdrawal thermal latency (e) in the ipsilateral hindpaw after intradermal injection. *n* = 10 per group, two‐way ANOVA, ^*^ *p* < 0.05, ^**^ *p* < 0.01 versus the Veh group. f,g) CRP‐induced mechanical (f) and thermal (g) hyperalgesia was alleviated in conditional knockout (CKO) rats. Changes in the paw withdrawal mechanical threshold (f) and the paw withdrawal thermal latency (g) in the ipsilateral hindpaw after intradermal injection. *n* = 7 per group, two‐way ANOVA, ^*^ *p* < 0.05, ^**^ *p* < 0.01 versus the littermate group. h,i) CRP‐sensitized naive rats to IgG‐immune complex (IgG‐IC) induced mechanical (h) and thermal (i) hyperalgesia. Changes in the paw withdrawal mechanical threshold (h) and the paw withdrawal thermal latency (i) in the ipsilateral hindpaw after intraplantar injection. *n* = 9 per group, two‐way ANOVA, ^*^ *p* < 0.05, ^**^ *p* < 0.01 versus the Veh group; ^##^ *p* < 0.01 versus the IgG‐IC + CRP group.

To investigate the cause of neuronal Fc*γ*RI/Syk signal activation after CCI, further experiments were performed to identify the neuronal Fc*γ*RI ligand. Previous studies found that CRP (also called PTX1) can bind and activate Fc*γ*RI.^[^ ^11^ ^]^ Our Western blot results revealed that nerve injury induced long‐lasting increased expression of CRP (endogenous ligand of Fc*γ*RI) in the DRG (**Figure** **6**a). qPCR results showed that the mRNA level of *Crp* continuously increased from 7 to 21 days after nerve injury in the DRG of rats (Figure S4a, Supporting Information). To determine the cellular distribution of CRP, we then performed double immunolabeling using PGP9.5 with CRP. The results showed that CRP was more colocalized with PGP9.5 in DRGs after nerve injury (Figure 6b,c). Immunostaining also indicated that CRP protein was coexpressed with Fc*γ*RI in the DRG neurons of rats (Figure S4b, Supporting Information). Furthermore, we directly determined whether CRP interacts with Fc*γ*RI. Co‐IP showed that the protein Fc*γ*RI was more strongly bound to CRP proteins (Figure S4c,d, Supporting Information).

To assess the behavioral effects of CRP, the intracutaneous injection of recombinant CRP was performed in the hind paws of naive rats. Intradermal injection of CRP induced dose‐dependent mechanical and thermal hyperalgesia in naive rats, whereas injection of vehicle (normal saline) did not evoke significant hyperalgesia compared with the baseline (Figure 6d,e). To investigate whether DRG neuronal Fc*γ*RI mediates CRP‐induced pain, recombinant CRP protein was injected into the hind paws of littermate control and CKO rats. The results revealed that CRP‐induced mechanical and thermal hyperalgesia was significantly alleviated in CKO rats (Figure 6f,g). Previous research revealed that IgG‐IC intracutaneous injection (1‐10 µg/ml, 20 µl) can induce a significant decrease in the mechanical withdrawal threshold or thermal withdrawal latency.^[^ ^6^ ^]^ Further research found that recombinant CRP significantly enhanced IgG‐IC intracutaneous injection‐induced pain in rats (Figure 6h,i).

**Figure 7:**
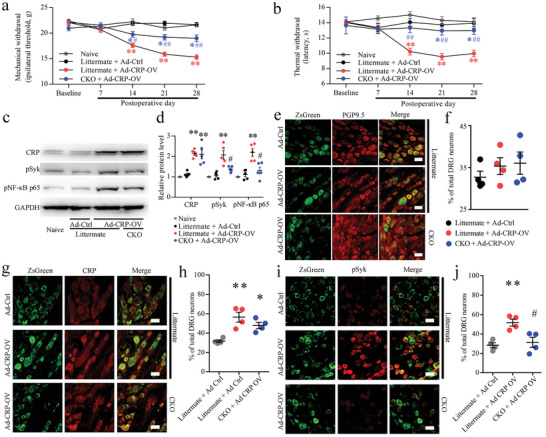
C‐reactive protein (CRP) overexpression induced pain and increased neuronal Fc‐gamma‐receptor type I (Fc*γ*RI) related neuroinflammation in dorsal root ganglions (DRGs). a,b) CRP overexpression in the DRG induced Fc*γ*RI‐mediated mechanical (a) and thermal hyperalgesia (b) in rats. Two‐way ANOVA, *n* = 9 per group, ^*^ *p* < 0.05, ^**^ *p* < 0.01, versus the naive group and littermate + Ad Ctrl group. ^#^ *p* < 0.05, ^##^ *p* < 0.01, versus littermate + Ad‐CRP‐OV. c) Western blot showing the expression levels of CRP overexpression and activation of Sykphosphorylated NF‐kappa B p65 subunit (NF‐*κ*B p65) signals in DRGs of littermate and conditional knockout (CKO) rats after DRG injection of AAV2/9‐hSyn‐CRP. d) Data summary of the expression levels of CRP, phosphorylated Syk (pSyk), and pNF‐*κ*B p65 in the DRGs of littermate and CKO rats after DRG injection of AAV2/9‐hSyn‐CRP. One‐way ANOVA, *n* = 5 per group, ^**^ *p* < 0.01 versus the naive group and littermate + Ad‐Ctrl group, ^#^ *p* < 0.05 versus the littermate + Ad‐CRP‐OV group. e) Coexpression of the ZsGreen tag with PGP9.5 in neurons after DRG injection of AAV2/9‐hSyn‐CRP. f) The positive rate of ZsGreen tag‐positive neurons in the littermate + Ad Ctrl group, littermate + Ad‐CRP‐OV group, and CKO + Ad‐CRP‐OV group. *n* = 4 rats per group. g) Coexpression of the ZsGreen tag with CRP after DRG injection of AAV2/9‐hSyn‐CRP. h) Data summary of CRP‐positive neurons in the DRGs of littermate and CKO rats after DRG injection of AAV2/9‐hSyn‐CRP. i) Coexpression of the ZsGreen tag with pSyk in neurons after DRG injection of AAV2/9‐hSyn‐CRP. j) Data summary of pSyk‐positive neurons in the DRGs of littermate and CKO rats after DRG injection of AAV2/9‐hSyn‐CRP. One‐way ANOVA, *n* = 4 per group, ^**^ *p* < 0.01 versus the littermate + Ad Ctrl group, ^#^ *p* < 0.05 versus the littermate + Ad‐CRP‐OV group. Scale bars: 50 µm (e,g,h).

To further assess the pain‐related function of CRP in DRG neurons, we overexpressed CRP in DRG neurons of rats by microinjection of the AAV2/9‐Syn‐CRP‐ZsGreen vector into L_4_‐L_5_ DRGs to encode CRP driven by the Syn promoter and establish a CRP overexpression model in vivo. Two weeks after injection of AAV, CRP overexpression produced long‐lasting mechanical and thermal hyperalgesia on the ipsilateral hind paw in littermate rats compared with naive rats or AAV2/9‐Syn‐null‐ZsGreen (Ad‐Ctrl) injection rats, and the CRP overexpression‐induced mechanical and thermal hyperalgesia was significantly alleviated in CKO rats (**Figure** **7**a,b). Western blot results further revealed that DRG injection of AAV2/9‐Syn‐CRP‐ZsGreen vector resulted in overexpression of CRP protein in the DRGs of littermate or CKO rats (Figure 7c,d). We then examined the effect of AAV2/9‐Syn‐CRP‐ZsGreen (Ad‐CRP‐OV) on Syk signal activation. Compared with the Ad‐Ctrl injection rats and naive rats, the injection of Ad‐CRP‐OV induced activation of Syk (pSyk) and NF‐*κ*B p65 (pNF‐*κ*B p65) in the DRG of littermate rats (Figure 7c,d). Further analysis showed that Ad‐CRP‐OV‐induced increases in pSyk and pNF‐*κ*B p65 levels were significantly decreased in the Ad‐CRP‐OV injection group of CKO rats (Figure 7c,d).

Meanwhile, double immunolabeling showed that L_4_‐L_5_ DRGs exhibited significant ZsGreen (green) labeling of neurons, resulting in an L_4_‐L_5_ DRG neuron (PGP9.5‐positive cells, red) labeling efficiency of 32.70% ± 1.45% in the Ad‐Ctrl injection group of littermate rats, 35.33% ± 2.02% in the Ad‐CRP‐OV injection group of littermate rats, and 36.04% ± 2.64% in the Ad‐CRP‐OV injection group of CKO rats (Figure 7e,f). Immunolabeling images showed that ZsGreen labeling of DRG neurons also expressed CRP after Ad‐CRP‐OV injection (Figure 7g). Compared to Ad‐Ctrl injection, pSyk‐positive neurons in the DRG were increased after injection of Ad‐CRP‐OV in littermate rats and significantly reduced in the DRG of CKO rats (Figure 7h,i).

### 2.5 Decreased Neuronal CRP Expression in the DRG Suppresses Nerve Injury Induced Neuropathic Pain and Neuroinflammation

**Figure 8. D:**
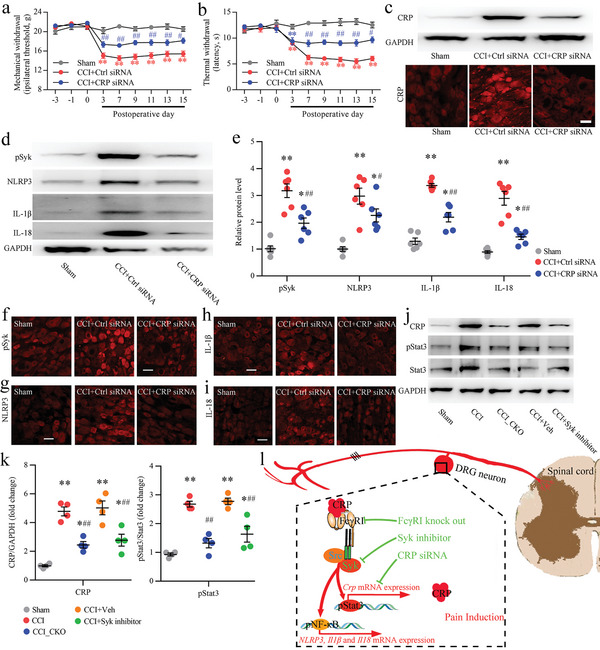
orsal root ganglion (DRG) injection of C‐reactive protein (CRP) siRNA inhibits nerve injury induced pain hypersensitivity and phosphorylated Syk (pSyk)/NLR family pyrin domain containing 3 (NLRP3) signaling activation. a,b) A preintraganglionic injection of CRP siRNA attenuated chronic constriction injury (CCI) induced mechanical allodynia (a) and thermal hyperalgesia (b). Ctrl siRNA is the control siRNA. Administration was performed 3 and 1 days before CCI operation. Two‐way ANOVA, *n* = 10 per group, ^*^ *p* < 0.05, ^**^ *p* < 0.01 versus the sham group, ^#^ *p* < 0.05, ^##^ *p* < 0.01, versus the CCI + Ctrl siRNA and the sham group. c) Western blot and immunostaining analysis of the effect of CRP siRNA on CRP protein levels and cellular expression in the DRG from CCI 15 d rats. d,e) Western blot analysis (d) and quantification (e) of the effect of CRP‐specific siRNA on pSyk, NLRP3, IL‐1*β*, and IL‐18 protein levels in the DRG from CCI rats 15 d after the operation. One‐way ANOVA, *n* = 6 per group, ^*^ *p* < 0.05, ^**^ *p* < 0.01 versus the sham group; ^#^ *p* < 0.05, ^##^ *p* < 0.01 versus the CCI + Ctrl siRNA. f–i) Immunostaining analysis of the effect of CRP‐specific siRNA on pSyk, NLRP3, IL‐1*β*, and IL‐18 cellular expression in DRG neurons from CCI rats 15 d after the operation. j,k) Western blot analysis (j) and quantification (k) of the effect of Fc*γ*RI/pSyk signaling inhibition on the CRP expression level and Stat3 phosphorylation level in the DRG from CCI rats 21 d after nerve injury. One‐way ANOVA, *n* = 4 per group, ^*^ *p* < 0.05, ^**^ *p* < 0.01 versus the sham group, ^#^ *p* < 0.05 versus the CCI group and the CCI + Veh group. l) Schematic illustration demonstrates CRP/Fc*γ*RI positive feedback proinflammatory signaling of neuropathic pain induced by nerve injury.

To determine the role of neuronal CRP in nerve injury induced neuropathic pain, we knocked down CRP expression with a specific siRNA in the L_4_‐L_5_ DRG of rats. We first evaluated the effect of CRP siRNA on nerve injury induced neuropathic pain and found that compared with CCI rats that were injected intraganglionic with siRNA control, injection intraganglionic with CRP siRNA significantly attenuated nerve injury induced neuropathic pain from POD7 until POD15 (**Figure** **8**a,b). To evaluate the effect of CRP siRNA on decreasing the CRP level, we performed intraganglionic injection of CRP siRNA into native rats. The expression level of CRP in the DRG was assessed by Western blot after intraganglionic injection of CRP siRNA. The results indicated that the expression level of CRP was decreased in the DRGs of rats after intrathecal injection with CRP siRNA compared with siRNA control rats (Figure 8c; Figure S4e, Supporting Information). Subsequently, our results found that intraganglionic injection with CRP siRNA significantly decreased the CCI‐induced activation level of Syk (pSyk level) and the expression levels of NLRP3, IL‐1*β*, and IL‐18 in the DRGs of rats with CCI POD15 (Figure 8d,e). Immunostaining results further found that the DRG of CCI rats revealed decreased expression levels of pSyk, NLRP3, IL‐1*β*, and IL‐18 in neurons after intraganglionic injection with CRP siRNA (Figure 8f–i).

A previous study reported that the transcription factor signal transducer and activator of transcription 3 (Stat3) is involved in mouse CRP gene expression.^[^ ^12^ ^]^ The activation of Stat3 plays a key role in the expression of CRP, and we found that the phosphorylation level of Stat3 in the DRG was increased after CCI (Figure 8j,k). We further investigated the potential effects of Fc*γ*RI/pSyk signaling on Stat3 activation and CRP protein levels in the DRG after CCI operation. In comparison to rats subjected to the CCI operation, *Fcgr1* CKO rats or rats injected with Syk inhibition with the CCI operation showed that the phosphorylation level of Stat3 and the expression level of CRP protein were significantly decreased in the DRG (Figure 8j,k). A schematic illustration of the proposed CRP/Fc*γ*RI positive feedback proinflammatory signaling of neuropathic pain is provided in Figure 8l. These results might suggest a potential strategy for the treatment of neuropathic pain targeting the CRP/Fc*γ*RI/Syk signaling pathway.

### 2.6 DRG Injection of Naive IgG Suppresses Persistent Pain and Neuroinflammation after CCI

**Figure 9:**
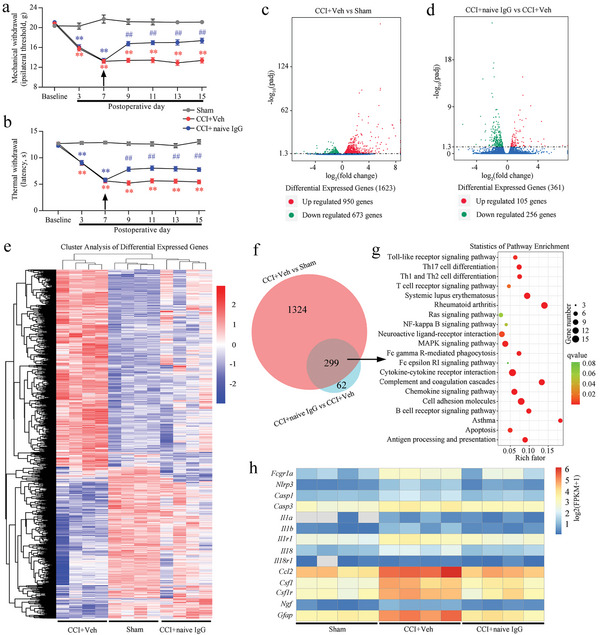
Inhibitory effects of naive IgG on nerve injury induced pain hypersensitivity after dorsal root ganglion (DRG) injection. a,b) Nerve injury induced mechanical allodynia (a) and thermal hyperalgesia (b) were attenuated in rats by naive IgG. Two‐way ANOVA, *n* = 14 per group, ^*^ *p* < 0.05, versus the sham; ^#^ *p* < 0.05, the chronic constriction injury (CCI) + naive IgG versus CCI. c) Volcano plot showing the overall distribution of upregulated and downregulated mRNAs between the sham group and the CCI + Veh group rats (*n* = 4 per group). d) Volcano plot showing the overall distribution of upregulated and downregulated mRNAs between the CCI + Veh group and CCI + naive IgG group (*n* = 4 per group). e) Heatmap of mRNAs showing hierarchical clustering of differentially expressed mRNAs in the three groups. In the clustering analysis, upregulated genes (red) and downregulated genes (blue) are shown. f) Venn diagram indicating the numbers of overlapping and distinct mRNAs between the CCI + Veh versus sham group and CCI + naive IgG versus CCI + Veh group. g) KEGG pathway scatterplot of genes associated with overlapping mRNAs from F. Rich factor, *q* value, and Gene number of enriched in this pathway were used to measure the enrichment degree of genes in the KEGG analysis. Rich factor refers to the number of differentially expressed genes (DEGs) enriched in a pathway as a ratio of the number of annotated genes. The rich factor value represents the enrichment degree. The *q* value represents a *p* value corrected by multiple hypothesis tests. h) Heatmap showing the differential expression of *Fcgr1* and proinflammatory cytokine genes in the DRGs of the sham, CCI + Veh and CCI + naive IgG rats.

In CRP‐overexpressing rats, which displayed enhanced hyperalgesia by CRP, we next performed DRG application of naive IgG to investigate the effect of analgesia in the CCI model of rats. The application of naive IgG gradually increased the nociceptive (mechanical and thermal) threshold evoked by the CCI operation (**Figure** **9**a,b). To evaluate the effects of naive IgG on gene expression in nerve‐injured DRGs, RNA sequencing was used to analyze the messenger RNA (mRNA) profiles in DRGs obtained from rats with sham operation, nerve injury (vehicle application), or nerve injury (naive IgG application). Compared with the sham group, the nerve injury group treated with vehicle had 473 differentially expressed genes (DEGs) in the injured DRGs (Figure 9c). A total of 361 DEGs, 105 upregulated and 256 downregulated, were identified in the nerve‐injured DRGs with naive IgG application compared with the nerve‐injured DRG group treated with vehicle (Figure 9d). A heatmap of the mRNA expression levels in the DRGs of the three groups (sham operation, CCI injury with vehicle and CCI injury with naive IgG) is shown in Figure 9e. We comparatively analyzed the overlapping mRNA of DEGs in nerve injury with vehicle group versus sham group and nerve injury with naive IgG group versus nerve injury with vehicle group DRGs. A Venn diagram was presented to depict the overlaps between the two sets of DEGs (Figure 9f). We further identified multiple signaling pathways relevant to both inflammation and immune regulation, such as the Fc*γ*R signaling pathway, cytokine–cytokine receptor interaction, chemokine signaling pathway, and Toll‐like receptor signaling pathway, by KEGG analysis (Figure 9g). mRNA sequencing revealed that the FPKM values of Fcgr1a and related proinflammatory cytokine genes in DRGs were increased after nerve injury and significantly decreased after naive IgG microinjection (Figure 9h).

**Figure 10:**
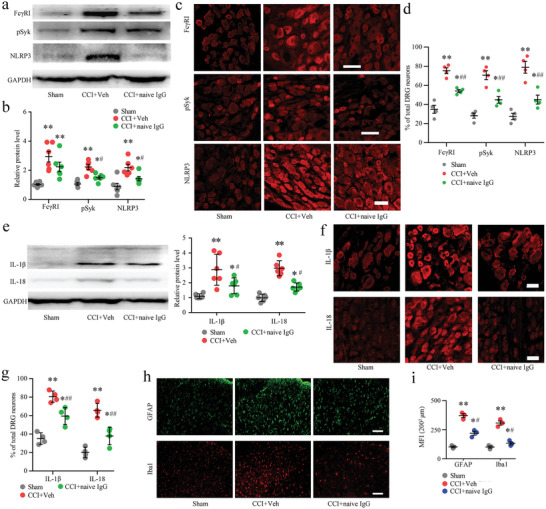
Naive IgG suppresses nerve injury induced Fc‐gamma‐receptor type I (Fc*γ*RI)/phosphorylated Syk (pSyk)/NLR family pyrin domain containing 3 (NLRP3) signaling activation after dorsal root ganglion (DRG) injection. a) Western blot analysis of the effects of naive IgG on the neuronal Fc*γ*RI/pSyk/NLRP3 signaling pathway in DRGs after chronic constriction injury (CCI) injury. b) Data summary of the protein expression levels of Fc*γ*RI, pSyk, and NLRP3 in the inhibitory effects of naive IgG on nerve injury induced rat DRGs after operation. *n* = 6 per group. ^*^ *p* < 0.05, ^**^ *p* < 0.01 versus the sham group; ^#^ *p* < 0.05 versus the CCI + Veh group by one‐way ANOVA. c) Immunostaining showing the Fc*γ*RI, pSyk, and NLRP3 cellular distribution of DRG neurons in sham, CCI + Veh and CCI + naive IgG rats. d) The positive rates of Fc*γ*RI, pSyk and NLRP3 in DRG neurons from sham, CCI + Veh and CCI + naive rats 15 d after the operation. One‐way ANOVA, *n* = 4 per group, ^*^ *p* < 0.05, ^**^ *p* < 0.01 versus the sham group; ^##^ *p* < 0.01 versus the CCI + Veh group. e) Western blot analysis of IL‐1*β* and IL‐18 expression in the DRGs of sham, CCI + Veh, and CCI + naive IgG rats. One‐way ANOVA, *n* = 6 per group, ^*^ *p* < 0.05, ^**^ *p* < 0.01 versus the sham group; ^##^ *p* < 0.05 versus the CCI + Veh group. f) Immunostaining showing the IL‐1*β* and IL‐18 cellular distribution of DRG neurons in sham, CCI + Veh and CCI + naive IgG rats. g) The positive rates of IL‐1*β* and IL‐18 in DRG neurons from the sham, CCI + Veh, and CCI + naive rats 15 d after the operation. *n* = 4 per group, by one‐way ANOVA, ^*^ *p* < 0.05, ^**^ *p* < 0.01 versus the sham group; ^##^ *p* < 0.01 versus the CCI + Veh group. h) Immunostaining showing the inhibitory effects of naive IgG on the activation of astrocytes (GFAP) and microglial cells (Iba1) in DH. i) Immunofluorescence intensity analysis shows the immunoreactivity of GFAP and Iba1 in the DH in the sham, CCI + Veh, and CCI + naive IgG rats. One‐way ANOVA, *n* = 3 per group, ^*^ *p* < 0.05, ^**^ *p* < 0.01 versus the sham group; ^#^ *p* < 0.05 versus the CCI + Veh group. Scale bars: 50 µm (c,f, h).

We further explored whether naive IgG attenuated neuropathic pain by suppressing neuronal Fc*γ*RI/Syk signal activation. We also examined the changes in NLRP3 proinflammatory signaling. Western blotting experiments revealed that DRG microinjection of naive IgG significantly reduced the expression level of Fc*γ*RI, phosphorylation level of Syk (pSyk), and expression level of NLRP3, which was induced by CCI (**Figure** **10**a,b). To investigate whether naive IgG microinjection blocks and attenuates CCI‐activated neuronal Fc*γ*RI/Syk/NLRP3 proinflammatory signaling in the DRG, immunofluorescence confirmed that DRG microinjection of naive IgG significantly reduced the activation of Syk/NLRP3 proinflammatory signaling induced by nerve injury in DRG neurons (Figure 10c,d). Meanwhile, the protein levels of the proinflammatory cytokines IL‐1*β* and IL‐18, which are regulated by NLRP3, were alleviated in the DRG neurons of CCI rats after DRG injection of naive IgG (Figure 10e). Then, the nerve injury induced cytokines IL‐1*β* and IL‐18 were mainly expressed in DRG neurons, and naive IgG injection suppressed IL‐1*β* and IL‐18 expression in the DRG (Figure 10f,g). Double immunofluorescence further confirmed that IL‐1*β* and IL‐18 protein immunoreactivity was distributed in the neurons (PGP9.5‐positive) of the DRG (Figure S5, Supporting Information). In the spinal dorsal horn, nerve injury induced activation of astrocytes (GFAP‐positive) and microglial cells (Iba1‐positive) was greatly suppressed by DRG injection of naive IgG (Figure 10h,i). These findings demonstrate that DRG injection of naive IgG can relieve nerve injury induced chronic pain by suppressing the activation of neuronal Fc*γ*RI‐related Syk signaling in DRGs.

## 3 Discussion

Our study reveals a critical role of neuronal CRP/Fc*γ*RI/Syk signaling in the neuropathic pain of nerve injury. Nerve injury induced neuronal Fc*γ*RI‐related Syk signaling activation may contribute to neuropathic pain after nerve injury by regulating the proinflammatory cytokines IL‐1*β* and IL18 in neurons in the DRG. Nerve injury also causes rapid and long‐lasting high expression of CRP in DRG neurons. CRP may be involved in the production and persistence of chronic pain in the CCI model of rats by activating the neuronal Fc*γ*RI‐related Syk signaling pathways of DRGs. DRG CKO or DRG naive IgG blockade of neuronal Fc*γ*RI alleviates nerve injury induced neuropathic pain. Meanwhile, inhibition of neuronal Fc*γ*RI suppresses nerve injury induced activation of the Syk/NF‐*κ*B p65 proinflammatory signaling pathway and induction of neuronal IL‐1*β* and IL‐18 in the DRG, as well as activation of astrocytes and microglial cells in the spinal dorsal horn. The results of our present study further confirm that Fc*γ*RI activation of sensory neurons may contribute to neuropathic pain. These findings may support a new understanding of the mechanism and therapy of neuropathic pain after nerve injury.

Fc*γ*Rs belong to the immunoglobulin superfamily and are classified as activating or inhibitory. In humans, Fc*γ*Rs include hFc*γ*RI, Fc*γ*RIIA, hFc*γ*RIIB, hFc*γ*RIIC, hFc*γ*RIIIA, and hFc*γ*RIIIB. Mice express four Fc*γ*Rs (I‐IV), and rats express three Fc*γ*Rs (I‐III).^[^ ^13^ ^]^ Activating Fc*γ*Rs contain immunoreceptor tyrosine activating motifs (ITAMs), and inhibitory Fc*γ*Rs contain immunoreceptor tyrosine inhibitory motifs.^[^ ^13c,d^ ^]^ Fc*γ*RI is known to be expressed most prominently on immune cells, including macrophages, dendritic cells, natural killer cells, neutrophils, eosinophils, and mast cells.^[^ ^13b–d^ ^]^ Beyond immune cells, recent studies have shown that neurons, astrocytes, and microglial cells of the central nervous system and peripheral nervous system also express Fc*γ*Rs, including Fc*γ*RI.^[^ ^5^, ^14^ ^]^ In addition, sensory neurons of the DRG express Fc*γ*RI, as reported by several studies.^[^ ^5a,b,d,e^ ^]^ Previous studies found that Fc*γ*RI in DRGs also contributes to joint pain in rodent models of RA. In autoimmune diseases such as RA, Fc*γ*RI, which is expressed on nociceptors of the DRG, is activated by autoantigen‐antibody‐IC.^[^ ^7^ ^]^ Autoantibodies have also been found to be a potential mechanism driving pain in complex regional pain syndrome (CRPS). Autoantibodies against voltage‐gated potassium channels can be detected in patient blood samples of CRPS. Many studies have found that autoimmunity plays a role in the initiation and maintenance of neuropathic pain.^[^ ^3c^ ^]^ Depletion of B cells may also be a disease‐modifying treatment for pain in a rodent model of CRPS.^[^ ^15^ ^]^ We showed that intraplantar injection of IgG‐IC rapidly induced mechanical and thermal hyperalgesia in wild‐type rats.

Fc*γ*RI is the only high‐affinity activating Fc*γ*R and binds to the Fc portion of IgG. Multimeric IgG‐IC cross‐links Fc*γ*RI to enable receptor clustering, aggregation, and activation, leading to ITAM domain phosphorylation. The recruitment and activation of Src and Syk kinases are crucial steps in the activation of Fc*γ*RI.^[^ ^16^ ^]^ Our previous study demonstrated that IgG‐IC activates neuronal Fc*γ*RI in DRGs by coupling with the TRPC3 cation channel through the Syk signaling pathway.^[^ ^10^ ^]^ Activating Syk further activates downstream signaling components, including Src,^[^ ^16b^ ^]^ Sos1,^[^ ^17^ ^]^ and Vav1,^[^ ^18^ ^]^ including NF‐*κ*B‐mediated transcription of cytokines.^[^ ^19^ ^]^ In a rat model of RA, the phosphorylation levels of Src and Syk were increased in DRG neurons.^[^ ^5c^ ^]^ In this study, several key molecules (Syk, Src, and NF‐*κ*B p65) in the Fc*γ*RI signaling pathway were found to be significantly regulated in DRG neurons of rats after nerve injury. We further found that CKO of neuronal Fc*γ*RI in the DRG or Syk inhibition can attenuate CCI‐induced Syk activation and NF‐*κ*B p65 activation in DRG neurons and decrease NLRP3, IL‐1*β*, and IL‐18 expression levels in DRG after neuropathic pain.

In addition to the interaction of Fc*γ*RI with IgG in passive immunization, Fc*γ*RI is also activated by CRP, which is an innate immunity‐related protein.^[^ ^20^ ^]^ The innate immune response is the first line of defense against sterile tissue damage and infection.^[^ ^21^ ^]^ CRP is a member of the pentraxin family, a ligand for Fc receptors (bound to Fc*γ*RI with 2–4 µµ affinity (dissociation constants *K*_D_)) on phagocytes and a major acute phase protein in tissue damage and inflammation.^[^ ^11^, ^22^ ^]^ CRP is unanimously regarded as an inflammatory biomarker associated with depression, schizophrenia, posttraumatic stress disorder, and autism.^[^ ^23^ ^]^ Previous studies have reported that CRP has a direct proinflammatory effect on endothelial cells and enhances IgG‐mediated phagocyte responses in immune thrombocytopenia.^[^ ^24^ ^]^ Elevated CRP levels in pain, including fibromyalgia/chronic pain syndrome^[^ ^25^ ^]^ and low back pain,^[^ ^26^ ^]^ have also been reported. In the present experiments, our results showed that neuronal CRP in the DRG was increased after nerve injury, and intraplantar injection of CRP produced mechanical and thermal hyperalgesia and aggravated IC‐induced pain in rats. CRP can induce the expression of pro‐IL‐1*β* and NLRP3 and activate the NLRP3 inflammasome in endothelial cells via the Fc*γ*R/NF‐kB pathway.^[^ ^27^ ^]^ Our previous work showed that mast cells are not involved in the pain induced by IgG‐IC.^[^ ^6^ ^]^ Previous studies on the chronic pain of arthritis have reported that immune cells are not necessary for IgG‐IC‐mediated joint pain. Conditional deletion of *Fcgr1* in sensory neurons significantly reduces IgG‐IC‐induced pain in arthritis models in mice and rats.^[^ ^5^, ^7^ ^]^ We observed that deletion of *Fcgr1* in sensory neurons of the DRG significantly alleviated CRP‐induced pain in *Fcgr1* CKO rats. CRP overexpression in DRG‐induced pain was also reduced in *Fcgr1* CKO rats. Stat3 regulates CRP gene expression and plays a key role in CRP expression.^[^ ^12^ ^]^ We found that suppression of the neuronal Fc*γ*RI/Syk pathway decreased the phosphorylation level of Stat3 and the expression level of CRP in the DRG after CCI operation.

Intravenous immunoglobulin (IVIG) is widely used in the immunotherapy of autoimmune and inflammatory diseases such as RA, Kawasaki disease, systemic sclerosis, Guillain–Barre syndrome, and chronic inflammatory demyelinating polyneuropathy.^[^ ^28^ ^]^ IVIG has been shown to be effective in treating inflammation‐related pain symptoms of rheumatic diseases. Previous research has revealed that IVIG may be beneficial for managing neuropathic pain.^[^ ^29^ ^]^ Some research has reported that IVIG and plasma exchange therapy could effectively improve pain symptoms in some CRPS patients.^[^ ^30^ ^]^ Fc*γ*RI, which is the high‐affinity Fc‐gamma receptor, can bind both IgG‐IC and monomeric IgG.^[^ ^11^, ^31^ ^]^ IgG_1_ bound to Fc*γ*RI with an affinity of 0.03 µµ, higher than CRP binding to other Fc*γ*Rs. We examined the therapeutic effect of naive IgG injection in the local DRG and found persistent pain relief in CCI rats. Naive IgG Fc alone has anti‐inflammatory or immunomodulatory activity.^[^ ^11^, ^31^, ^32^ ^]^ A high level of monomeric IgG can bind Fc*γ*RI and block or reduce the IC‐mediated activation of Fc*γ*RI.^[^ ^16^, ^31^, ^32^ ^]^ Based on nociceptive neurons of the DRG expressing Fc*γ*RI, we found that IgG injection of local DRG reduced the local neuroinflammation of DRG by modulating neuronal Fc*γ*RI/Syk signaling. Activation of microglia^[^ ^33^ ^]^ and astrocytes^[^ ^34^ ^]^ plays a critical role in the development and maintenance of neuropathic pain. The expression of Iba1 (microglia cell marker) and GFAP (astrocyte marker) was increased after neuropathic pain.^[^ ^35^ ^]^ Activation of microglia and astrocytes participates in central sensitization and neuroinflammation in the spinal cord after neuropathic pain.^[^ ^33^, ^34^, ^35^ ^]^ We found that IgG injection significantly attenuated neuropathic pain 15 days after nerve injury, while neuropathic pain‐induced elevated Iba1 and GFAP expression levels were decreased in the spinal cord after IgG injection. These results revealed that the activation of glial cells and central sensitization after neuropathic pain are suppressed by IgG injection.

## 4 Conclusions 

In summary, the present study supports a novel view of how nerve injury induced CRP and Fc*γ*RI mediate the nociceptive behavior of neuropathic pain. Local formation of naive IgG has the potential to serve important roles in the therapy of chronic pain. Taken together, these results suggest that neuronal CRP/Fc*γ*RI positive feedback proinflammatory signaling plays an important role in neuropathic pain. These findings may provide novel insights into the interactions between nerve damage and peripheral neuroimmunity in pathologic conditions.

## 5 Experimental Section

### Animals

Male Sprague–Dawley rats were purchased from the National Institutes for Food and Drug Control (China). *Pirt* ^Cre/+^;*Fcgr1a^loxP/loxP^* (*Fcgr1a* CKO, maintained on a Sprague–Dawley genetic background) rats were described previously.^[^ ^5c^ ^]^ Littermate rats were generated by interbreeding heterozygotes on the Sprague–Dawley genetic background. Male rats weighing 150–180 g were used, and rats were raised in standard cages (five per cage) in a 12‐h light/dark cycle and climate‐controlled room with a specific pathogen‐free environment. Animals were randomly assigned to treatment or control groups. All animal procedures performed in this study were reviewed and approved by the Institutional Animal Care and Use Committee of the Institute of Basic Medical Sciences, Chinese Academy of Medical Sciences, Peking Union Medical College (Beijing, China) and were conducted in accordance with the guidelines of the International Association for the Study of Pain.

### Drugs and Drug Administration

The recombinant CRP protein (Cat: 80041‐R08H) was purchased from Sino Biological Inc.; ovalbumin (OVA, Cat: A5503) from Sigma–Aldrich; rat anti‐OVA IgG (Cat: ACMOV111R) from Agro‐Bio; highly selective Syk inhibitor PRT062607 (P505‐15) HCl (Cat: S8032) from Selleck; naive rat gamma globulin (Cat: 012‐000‐002, Jackson ImmunoResearch) which was obtained and purified from nonimmunized and healthy rats sera and nonspecific IgG that does not bind specifically to a specific target protein. AAV2/9‐hSyn‐CRP‐ZsGreen and AAV2/9‐hSyn‐ZsGreen (control vector) were made by Hanbio (China). As previously described^[^ ^5^, ^6^ ^]^ IgG‐IC was prepared by using OVA as the antigen and rat anti‐OVA IgG as the antibody. CRP protein was dissolved in 20 µL phosphate buffered saline (PBS). Rats were randomly divided into groups and injected intradermally into the plantar skin of the hindpaw with a 20 µl volume of CRP, IgG‐IC or PBS (vehicle).

### Intraganglionic Injection of rat DRG

As previously described,^[^ ^36^ ^]^ rats were anesthetized with pentobarbital sodium (40 mg kg^−1^ i.p.). Exposing the L_4_‐L_5_ spinal nerve, a microinjection syringe was inserted into the L_4_‐L_5_ spinal nerve under the epineurium until the tip reached the center of the DRG. Then, the drugs (5 µL of AAV vectors consisting of 10^12^ viral particles or naive IgG from rats) were delivered to the L_4_‐L_5_ DRGs.

### Rat Model of Neuropathic Pain

CCI rats were obtained according to the Bennett and Xie model.^[^ ^8^ ^]^ The sodium pentobarbital (40 mg kg^−1^ i.p.) was used to anesthetize rats and 4‐0 surgical catgut was used to loosely tie four ligatures around the sciatic nerve of the mid‐thigh level on the right side with approximately 1 mm space between the knots. Rats in the sham group only received sciatic nerve exposure without ligation.

### Behavioral Tests of Pain

As previously described,^[^ ^35^, ^37^ ^]^ mechanical and thermal hyperalgesia in rats was quantified. Experimental rats were randomly divided into groups, and researchers were blinded regarding the group allocation. Briefly, rats were placed in individual plastic cages (10 × 20 × 20 cm) on a mesh floor and allowed to acclimate for 30 min. Rats were acclimatized for three consecutive days before the behavioral test, and three measurements were averaged for each rat at 5–10 min intervals. The paw withdrawal threshold in response to mechanical stimuli was used to assess mechanical allodynia. The probe of an electric von Frey anesthesia meter (IITC Life Science) was applied perpendicularly to the hindpaw with no acceleration at a force. Acute withdrawal of the hindpaw was considered a positive response. Paw withdrawal latency in response to radiant heat was used to evaluate thermal hyperalgesia. The radiant heat source of a thermal stimulator (BME‐410C Plantar Test Apparatus) was focused on the hindpaw plantar surface of rats, which were positioned on the floor of the cage. When the rat moved or licked the hindpaw, the thermal stimulus was terminated, and the time from initiation to termination was recorded. A maximum cut‐off time of 20 s was applied to prevent tissue damage in the thermal stimulus tests.

### Immunoprecipitation and Immunoblotting

L_4_‐L_5_ DRGs were harvested from rats and snap‐frozen in liquid nitrogen. Total proteins were extracted with RIPA lysis buffer (Thermo Fisher Scientific) containing NP‐40, protease inhibitors (CoWin Biosciences), and phosphatase inhibitors (CoWin Biosciences). According to the protocol of the Pierce Crosslink Magnetic IP/Co‐IP Kit (Fisher Scientific, #88 805), DRG tissue lysates were immunoprecipitated over 2 h at 4 °C with protein A/G coupled with antibodies (rabbit anti‐Fc*γ*RI 1:1000, Cat: 80016‐R015, Sino Biological Inc.; rabbit anti‐Syk 1:1000, Cat: ab40781, Abcam; rabbit anti‐CRP 1:1000, Cat: ab259862, Abcam or control negative antibody (Cat: 3900, Cell Signaling Technology)). Proteins were resolved by 10% SDS–PAGE, transferred onto PVDF membranes (Merck Millipore), and immunoblotted with primary antibodies.

As previously described,^[^ ^35^, ^37^ ^]^ all homogenized samples of lumbar DRGs were centrifuged, and supernatants were mixed with SDS–PAGE loading buffer for 5 min at 95 °C. Thirty micrograms of each protein sample was separated by 10% SDS–PAGE and then transferred to PVDF membranes. All membranes were blocked with 5% BSA in TBST for 1 h at room temperature and subsequently incubated with rabbit anti‐Fc*γ*RI (1:1000, Cat: 80016‐R015, Sino Biological Inc.), rabbit anti‐FcR*γ* (1:500, Cat: ab151986, Abcam), rabbit anti‐Syk (1:1000, Cat: ab40781, Abcam), rabbit anti‐pSyk (1:1000, Cat: PA5‐36692, Thermo Fisher Scientific), rabbit anti‐pSrc (1:1000, Cat: ab185617, Abcam), rabbit anti‐Src (1:1000, Cat: ab109381, Abcam), rabbit anti‐CRP (1:500, Cat: ab259862, Abcam), rabbit anti‐Vav1 (1:500, Cat: D155205, Sangon Biotech), mouse anti‐pNF‐*κ*B p65 (1:500, Cat: 13 346, Cell Signaling Technology), rabbit anti‐NLRP3 (1:1000, Cat: ab263899, Abcam), rabbit anti‐IL‐18 (1:1000, Cat: PAB16177, Abnova), rabbit anti‐IL‐1*β* (1:1000, Cat: ab254360, Abcam), mouse anti‐Stat3 (1:1000, Cat: 9139, Cell Signaling Technology), rabbit anti‐Stat3 (1:1000, Cat: 9145, Cell Signaling Technology), mouse anti‐CRP (1:1000, Cat: 66250‐1‐Ig, Proteintech Group, Inc.), or mouse anti‐GAPDH (1:2000, Cat: ab8245, Abcam) primary antibodies. The corresponding secondary antibodies (HRP‐conjugated goat anti‐rabbit or goat anti‐mouse, 1:5000, CoWin Biosciences) and HRP‐conjugated VeriBlot for IP Detection Reagent (1:2000, Cat: ab131366, Abcam) were probed for 1 h at room temperature. The results were detected with an enhanced chemiluminescence reagent eECL Kit (Cat: CW0049, CoWin Biosciences).

### Immunofluorescence Staining

Fresh 4% paraformaldehyde was perfused into rats deeply anesthetized with sodium pentobarbital (40 mg kg^−1^) through the ascending aorta. The L_4_‐L_5_ DRGs and spinal dorsal horn were harvested, postfixed in 4% paraformaldehyde for 4 h and then dehydrated in 30% sucrose overnight at 4 °C. The frozen tissue was sectioned to a thickness of 12 µm in a cryostat. After permeabilization with 0.2% Triton X‐100 in PBS for 15 min and incubation with blocking buffer (10% normal donkey serum) for 1 h, the tissue sections were incubated with primary antibodies, such as rabbit anti‐Fc*γ*RI (1:500, Cat: 80016‐R015, Sino Biological Inc.), goat anti‐FcR*γ* (1:100, Cat: sc‐33496, Santa Cruz Biotech), rabbit anti‐pSyk (1:100, Cat: PA5‐36692, Thermo Fisher Scientific), rabbit anti‐Syk (1:100, Cat: ab40781, Abcam), rabbit anti‐Src (1:100, Cat: ab109381, Abcam), rabbit anti‐CRP (1:200, Cat: ab259862, Abcam), rabbit anti‐Vav1 (1:100, Cat: D155205, Sangon Biotech), rabbit anti‐Sos1 (1:200, Cat: ab140621, Abcam), mouse anti‐pNF‐*κ*B p65 (1:100, Cat: 13 346, Cell Signaling Technology), rabbit anti‐NLRP3 (1:200, Cat: ab263899, Abcam), rabbit anti‐IL‐18 (1:100, Cat: PAB16177, Abnova), rabbit anti‐IL‐1*β* (1:100, Cat: ab254360, Abcam), mouse anti‐CRP (1:100, Cat: 66250‐1‐Ig, Proteintech Group, Inc.), mouse anti‐Syk (1:100, Cat: MA1‐19332, Thermo Fisher Scientific), guinea pig anti‐TRPV1 (1:800, Cat: ab10295, Abcam), and guinea pig anti‐PGP9.5 (1:200, Cat: ab10410, Abcam) in PBS with 10% normal donkey serum overnight at 4 °C. Afterwards, the slides were incubated with the proper secondary antibodies (Alexa Fluor 594‐conjugated donkey anti‐rabbit, 1:600; Alexa Fluor 594‐conjugated donkey anti‐ guinea pig, 1:600; Alexa Fluor 488‐conjugated donkey anti‐mouse, 1:600 and Alexa Fluor 488‐conjugated donkey anti‐guinea pig, 1:600, Jackson ImmunoResearch) or Alexa Fluor 488‐conjugated IB4 (1:200, Cat: I21411, Thermo Fisher Scientific) for 1 h. In a separate experiment, control staining was performed (for blocking by adding protein of Fc*γ*RI (1:100, Sino Biological) with the Fc*γ*RI primary antibody) to test Fc*γ*RI primary antibody specificity. No primary antibody staining was also performed to test primary antibody specificity. Slides were then washed in PBS and cover‐slipped with VECTASHIELD Mounting Medium with DAPI (Cat: H‐1200, Vector lab). Images were captured by a microscopic imaging system (Olympus BX61 and FluoView software), and the percentages of positive neurons were calculated and statistically analyzed.

### RNA Sequencing

As previously described,^[^ ^5c^ ^]^ L_4_ to L_5_ lumbar DRGs were obtained from four rats in the sham group (15 days after operation), four rats in the CCI + Veh group (15 days after operation), and four rats in the CCI + IgG group (15 days after operation). Total RNA was extracted using TRIzol reagent (Thermo Fisher Scientific). RNA degradation and contamination were monitored on 1% agarose gels. RNA integrity was assessed using the RNA Nano 6000 Assay Kit of the Agilent Bioanalyzer 2100 system (Agilent Technologies, USA). Sequencing libraries were generated using the NEBNext UltraTM RNA Library Prep Kit for Illumina (NEB, USA) following the manufacturer's recommendations. The clustering of the index‐coded samples was performed on a cBot Cluster Generation System using TruSeq PE Cluster Kit v4‐cBot‐HS (Illumina) according to the manufacturer's instructions. After cluster generation, the library preparations were sequenced on an Illumina HiSeq 4000 platform, and paired‐end 150 bp reads were generated. Raw data (raw reads) in fastq format were first processed through in‐house Perl scripts. In this step, clean data (clean reads) were obtained by removing reads containing adapters, reads containing poly‐N sequences and low‐quality reads from the raw data. At the same time, the Q20, Q30, GC content, and sequence duplication level of the clean data were calculated. All downstream analyses were based on clean data with high quality. Gene function was annotated based on the following databases: Nt (NCBI nonredundant nucleotide sequences); KO (KEGG Orthology database); and GO (Gene Ontology). Quantification of gene expression levels was estimated by fragments per kilobase of transcript per million fragments mapped.

### Statistical Analysis

Data values are expressed as the group mean ± SEM. Statistical analyses were performed using SPSS software (version 17.0). Student's *t* test was used to analyze the statistical significance of differences between two groups. One‐way analysis of variance (ANOVA) followed by Scheffe's post‐hoc test was used to determine statistical comparisons of differences among three or more groups. Two‐way ANOVA followed by the Bonferroni post hoc test was used to determine significant differences in pain behavior. *p* < 0.05 was considered statistically significant. Differential expression analysis of the two groups was performed using the DESeq R package. Genes with an adjusted *p* value of less than 0.05 according to the DESeq analysis were defined as differentially expressed.

## Conflict of Interest

The authors declare no conflict of interest.

## Author Contributions

F.L., L.Z., and S.S. contributed equally to this work. F.L., Y.X., and C.M. designed the study. F.L., L.Z., S.S., Y.F., X.Y., and H.C. analyzed the data. F.L., L.Z., S.S., and J.S. performed the experiment. F.L., L.Z., and S.S. wrote the manuscript. All authors read and approved the final manuscript.

## Study approval

All animal studies were approved by the Institutional Animal Care and Use Committee of the Institute of Basic Medical Sciences Chinese Academy of Medical Sciences, Peking Union Medical College (#011‐2014 and #ACUC‐A01‐2021‐001).

## Supporting information

Supporting Information

## Data Availability

The data that support the findings of this study are available in the supplementary material of this article.
