# Supplementary material for: Neuronal C‐Reactive Protein/FcγRI Positive Feedback Proinflammatory Signaling Contributes to Nerve Injury Induced Neuropathic Pain
Source: Adv Sci (Weinh). 2023 Feb 2;10(10):2205397. doi: 10.1002/advs.202205397 (PMC10074098; doi:10.1002/advs.202205397)
Supplement: Supplementary file 1 — Supporting Information [file ADVS-10-2205397-s001.pdf]

Supporting Information

Supplementary Methods

Rat genotyping

The *Fcgr1* CKO (*Pirt*$^{CRE+}$;*Fcgr1*$^{loxP+/+}$) rat genotype was identified by PCR. Genomic DNA was prepared from 5 mm clipped tail samples from rats and extracted in DNA lysis buffer containing proteinase K using the Tail Genomic DNA Kit (Cat: CW2049S, CoWin Biosciences). The DNA solution of the rat genotype was identified by PCR with 2×Taq MasterMix (Cat: CW0682S, CoWin Biosciences). DNA was predenaturated at 94°C for 2 mins, followed by PCR with 30 cycles of 94°C for 30 s, 50°C for 30 s, and 72°C for 30 s. The primers for *Fcgr1loxP* were as follows: forwards, 5′-CTGTAATTCTGCTACTGTTATGAATCGTAATG-3′ and reverse, 5′-CCTGAACACCAAAATCTGCCTG-3′. The primers for *Pirt-Cre* were as follows: forwards, 5′-TACTGACGGTGGGAGAATG-3′ and reverse, 5′-CTGTTTCACTATCCAGGTTACG-3′.

Fluorescent in situ hybridization

To examine the expression of *Fcgr1* mRNA in DRG neurons of rats, fluorescent in situ hybridization (FISH) was used with locked nucleic acid probes specific for *Fcgr1*. Rats were sacrificed under anesthesia. The harvested DRGs were fixed with 4% paraformaldehyde. Protease K (20 µg/ml) was added to digest the frozen DRG sections at 37°C for 5 minutes. After washing with pure water, the sections were washed 3 times for 5 min each with PBS. After preincubation in hybridization solution at 37°C for 1 h, the sections were incubated overnight in hybridization solution with 8 ng/µl probes at 37°C. DAPI was added to the sections and incubated in the dark for 8 min. After rinsing, anti-fluorescence quenching sealant was added to the sections, which were then sealed. The probes were synthesized by Sangon Biotech (Shanghai, China) Co., Ltd. The sequence of *Fcgr1* mRNA probes (Cat: a263121): 5'Cy3-TTGCCCACCAACTGGAACCCAAAGTA-3', the sequence of *Fcgr1* exon 3 mRNA probes (Cat: a263122): 5'FITC-CACCACUGUCCUUGAAACUGGCCUUGAGGAUGC-3', the sequence of *Crp* mRNA probes (Cat: a263120): 5'FITC- AGGUGGCACAGAUGUGUGUUGGUACCUCAG-3'.

Quantitative real time PCR

L_4-L_5 DRGs were harvested from rats and flash-frozen in liquid nitrogen. Total RNA was extracted using TRIzol reagent (Invitrogen) and reverse transcribed using PrimeScriptTM RT Master Mix (Takara, Japan) according to the manufacturer's instructions. Quantitative RT-PCR (qPCR) was performed on a Bio-Rad CFX96 machine using SYBR Premix Ex Taq (Takara, Japan). The primers used were as follows: *Fcgr1* mRNA forwards, 5'-AGT TGG AGC TAT TTG GTC CCC AGT C-3'

and reverse, 5’- GCT AAG GTC CAG GGT CAC CTG A-3’; *Fcrg* mRNA forwards, 5’-CGC AGC TCT GCT ATA TCC TGG ATG-3’, and reverse, 5’-CTC ACG GCT GGC TAT GTC TGC-3’; *Crp* mRNA forwards, 5’- TGA GGC ACC TCC TGG GAT TA-3’ and reverse, 5’-TGC ACA TGC TGG AAG CCT TA-3’; *actin, beta (Actb)* mRNA forwards, 5’-GGA GTA GCA GCC AAT CTC TGT-3’ and reverse, 5’-TTG CCA GCA CCA CTC TGA CCG AA-3’. The expression levels of the target genes were quantified relative to the level of *Actb* gene expression using the 2ΔΔCT method. Real-time PCR experiments for each gene were performed on three separate occasions.

Supplementary Figures

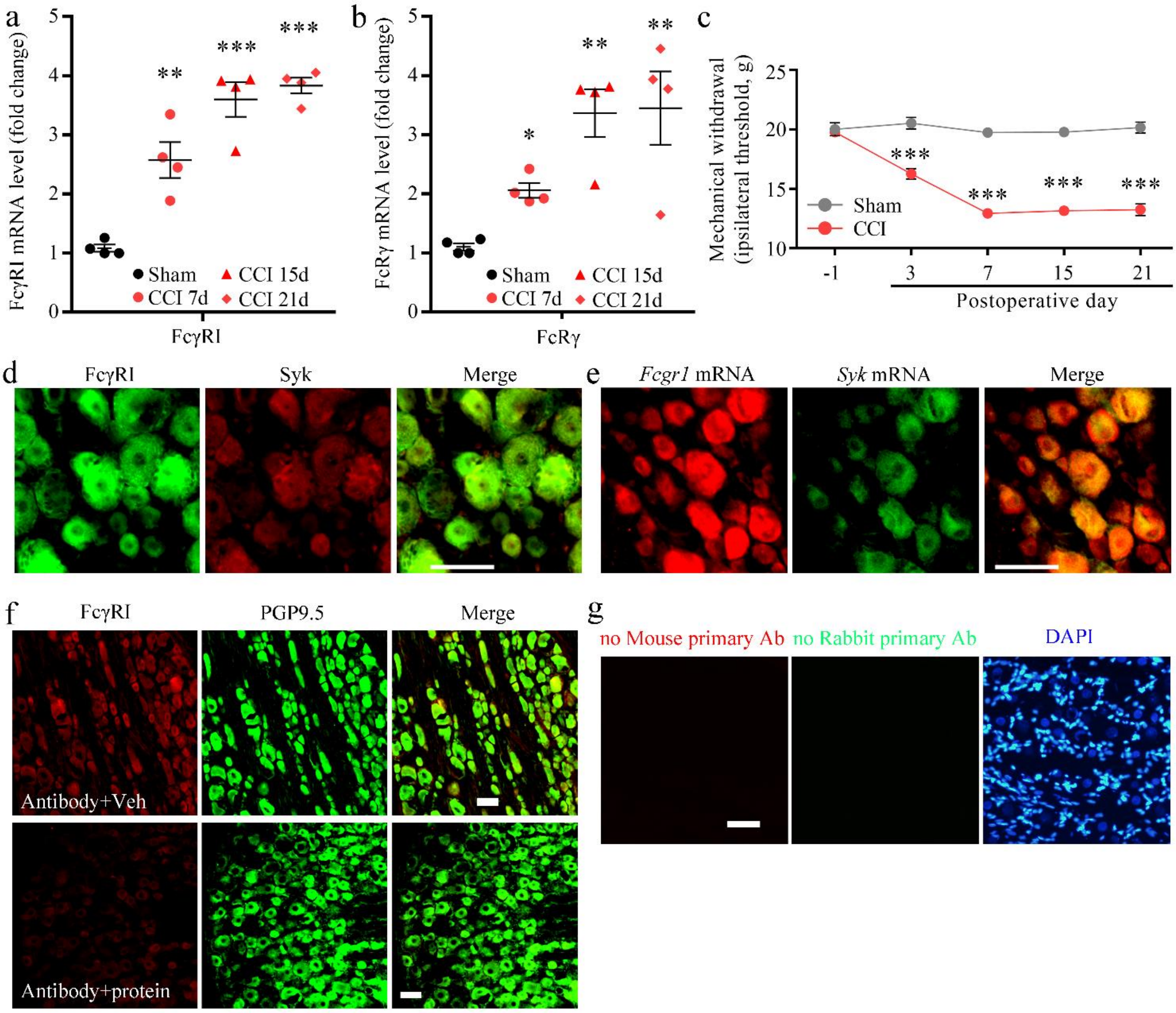

Figure S1. a, b) Time course of *Fcgr1* (a) and *Fcrg* (b) mRNA levels in DRG after CCI operation (n = 4 rats per group), determined by real-time PCR. $^{**}p < 0.01$, $^{***}p < 0.001$ versus the sham group, by one-way ANOVA. **c)** CCI-induced mechanical hypersensitivity manifested as a lowered threshold of thermal withdrawal. n = 10-26/group, $^{***}p < 0.001$ vs the sham group by two-way ANOVA. **d)** Double immunostaining showing cellular colocalization of FcγRI (green) and FcRγ (red) in rat DRGs from the sham groups. **e)** Double FISH analysis using *Fcgr1* (green) and Syk (red) mRNA expression in DRGs from sham groups. **f)** The blocking protein FcγRI was added with the primary antibody to test its specificity. The top row shows the FcγRI antibody with PBS, and the bottom row shows the FcγRI antibody with FcγRI protein. **g)** No primary antibody was used to test the primary antibody specificity. In d-g, scale bars = 50 μm.

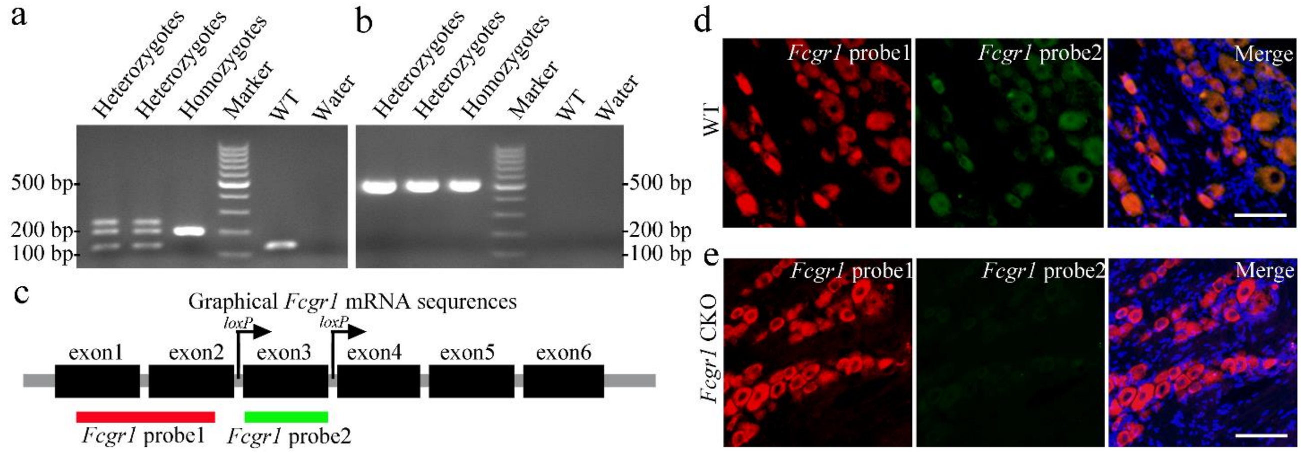

Figure S2. a) PCR analysis of the genotypes *loxP-flanked Fcgr1* in the CKO rats and WT rats. **b)** RT–PCR analysis of *Pirt-Cre* expression in CKO rats and WT rats. **c)** Diagram showing *Fcgr1* mRNA targets identified with fluorescence in situ hybridization (FISH) probe 1 (red) and FISH probe 2 (green). **d, e)** FISH analysis using the *Fcgr1* probe, revealing *Fcgr1* mRNA exon 3 expression (green) on DRGs from WT rats (d) and CKO rats (e). Homozygotes: $Pirt^{CRE+}$;$Fcgr1^{loxP+/+}$ (*Fcgr1* CKO) rats, Heterozygotes: $Pirt^{CRE+}$;$Fcgr1^{loxP+/+}$ rats, WT: wild-type rats. In d-e, scale bars = 100 μm.

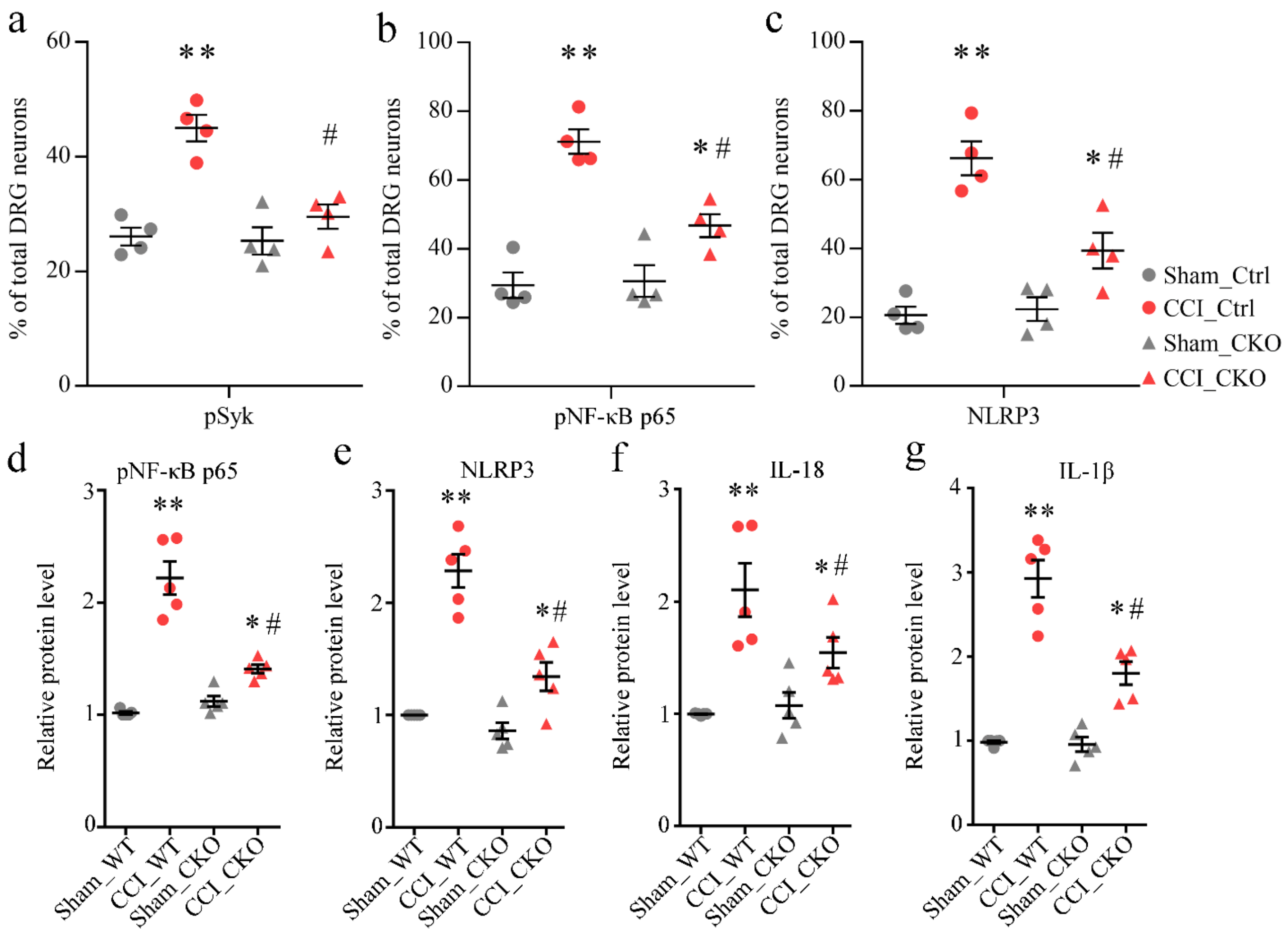

Figure S3. a) The positive rate of pSyk in the sham and CCI DRGs from littermate control (Ctrl) and CKO rats 21 d after surgery. **b)** The positive rate of pNF-κB p65 in the sham and CCI DRGs from Ctrl and CKO rats 21 d after surgery. **c)** The positive rate of NLRP3 in the sham and CCI DRGs from Ctrl and CKO rats 21 d after surgery. One-way ANOVA, n = 4 rats/group, *$p < 0.05$, **$p < 0.01$ vs the Sham_Ctrl group and Sham_CKO group; #$p < 0.05$ vs the CCI_Ctrl group. **d-g)** Data summary of the protein expression levels of pNF-κB p65 (d), NLRP3 (e), IL-18 (f) and IL-1β (g) in the sham and CCI DRGs from Ctrl and CKO rats 21 d after surgery. One-way ANOVA, n = 5 rats/group. *$p < 0.05$, **$p < 0.01$ versus the Sham_Ctrl group and Sham_CKO group, #$p < 0.05$ versus the CCI_Ctrl group.

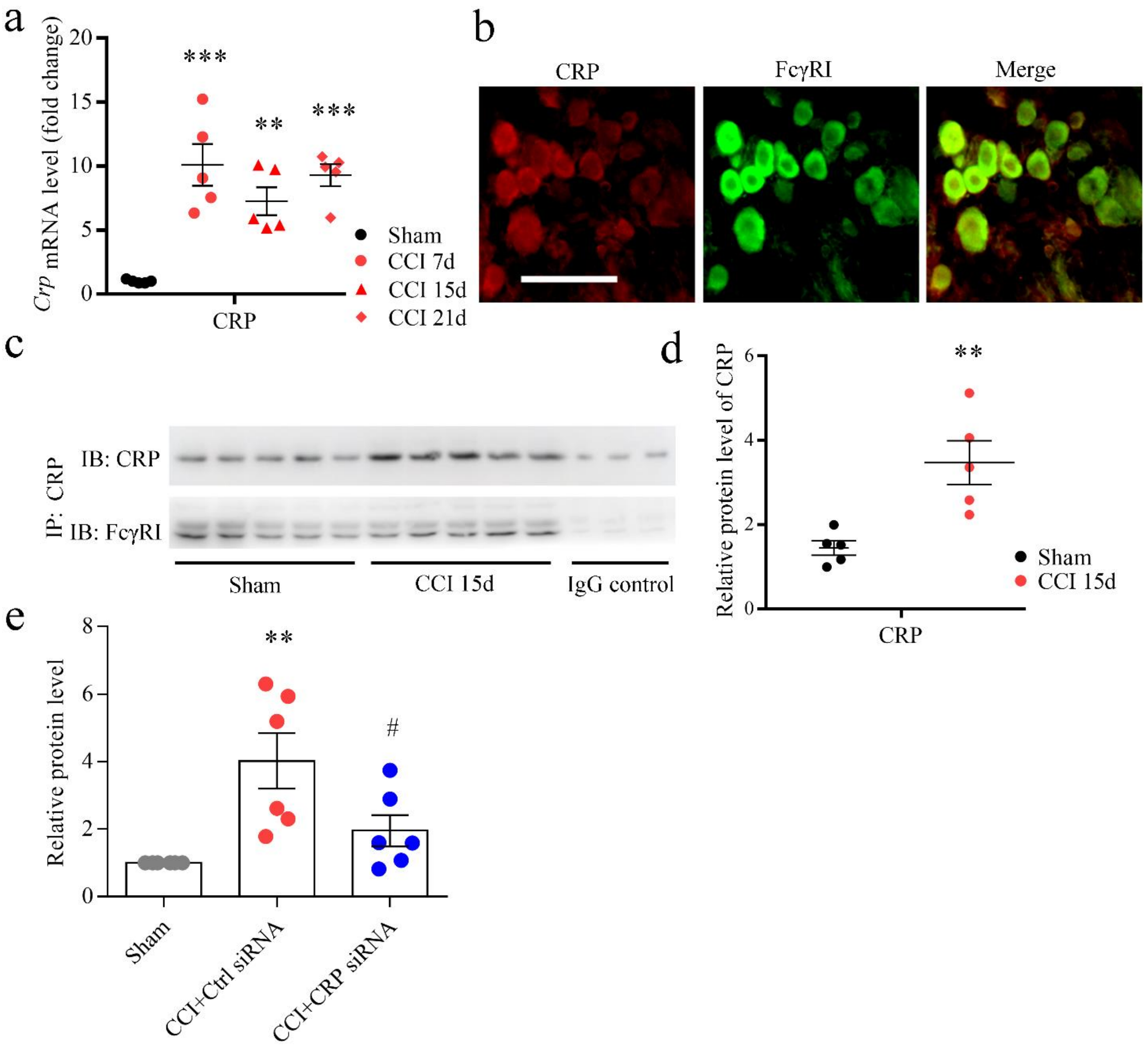

Figure S4. a) Time course of *Crp* mRNA levels in DRG after CCI operation (n = 5 rats per group), determined by real-time polymerase chain reaction. $^{**}p < 0.01$, $^{***}p < 0.001$ versus the sham group, n = 5/group, by one-way ANOVA. **b)** Double immunostaining showing cellular colocalization of CRP (red) and FcγRI (green) in rat DRGs from the sham groups. Scale bars = 50 µm **c, d)** Co-IP showed the interaction between FcγRI and CRP in DRG tissue from sham and CCI rats 15 d after surgery. n = 5/group, $^{**}p < 0.01$ vs the sham group, by Student's t-test. **e)** Quantification of the effect of CRP siRNA on CRP protein levels in DRGs from CCI 15 d rats. n = 6/group, $^{**}p < 0.01$ vs the sham group, $^{\#}p < 0.05$ vs the CCI+Ctrl siRNA group, by one-way ANOVA.

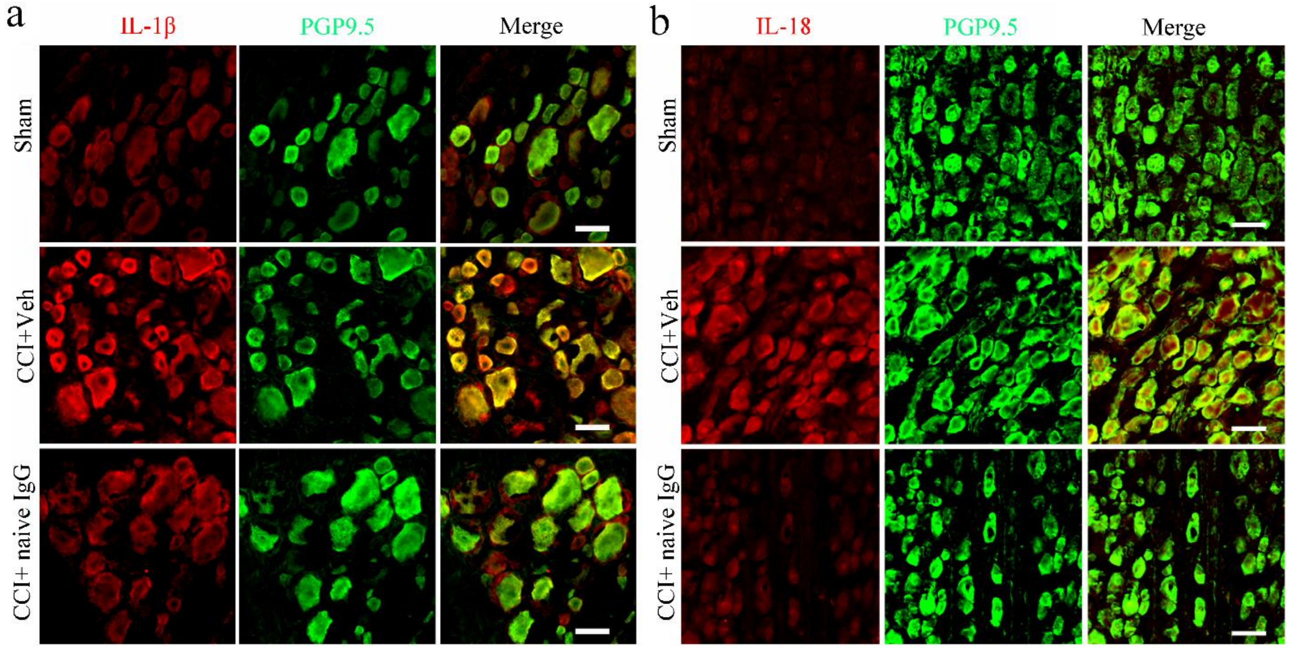

Figure S5. a) Double immunofluorescence for the cellular expression of IL-1β in the DRGs of the sham, CCI+Veh and CCI+IgG groups. Double immunostaining images showed IL-1β protein (red) and the neuronal marker PGP9.5 protein (green). **b)** Double immunofluorescence for cellular expression of IL-18 in DRGs of sham, CCI+Veh and CCI+IgG groups. Double immunostaining images showed IL-18 protein (red) and the neuronal marker PGP9.5 protein (green). Scale bars: 50 μm in a and b.
